# Simultaneous Assessment of Left Atrial Fibrosis and Epicardial Adipose Tissue Using 3D Late Gadolinium Enhanced Dixon MRI

**DOI:** 10.1002/jmri.28100

**Published:** 2022-02-07

**Authors:** Iulia Skoda, Markus Henningsson, Sofia Stenberg, Jonathan Sundin, Carl‐Johan Carlhäll

**Affiliations:** ^1^ Department of Cardiology in Linköping Linköping University Linköping Sweden; ^2^ Unit of Cardiovascular Sciences, Department of Health, Medicine and Caring Sciences Linköping University Linköping Sweden; ^3^ Center for Medical Image Science and Visualization (CMIV) Linköping University Linköping Sweden; ^4^ Department of Clinical Physiology in Linköping Linköping University Linköping Sweden

**Keywords:** 3D Dixon MRI, late gadolinium enhancement, atrial fibrillation, left atrial fibrosis, epicardial adipose tissue

## Abstract

**Background:**

Epicardial adipose tissue (EAT) may induce left atrium (LA) wall inflammation and promote LA fibrosis. Therefore, simultaneous assessment of these two important atrial fibrillation (AF) risk factors would be desirable.

**Purpose:**

To perform a comprehensive evaluation of 3D Dixon water–fat separated late gadolinium enhancement (LGE‐Dixon) MRI by analysis of repeatability and systematic comparison with reference methods for assessment of fibrosis and fat.

**Study Type:**

Prospective.

**Population:**

Twenty‐eight, 10, and 7 patients, respectively, with clinical indications for cardiac MRI.

**Field Strength/Sequence:**

A 1.5‐T scanner, inversion recovery multiecho spoiled gradient echo.

**Assessment:**

Twenty‐eight patients (age 58 ± 19 years, 15 males) were scanned using LGE‐Dixon. A 5‐point Likert‐type scale was used to grade the image quality. Another 10 patients (age 46 ± 19 years, 9 males) were scanned using LGE‐Dixon and 3D proton density Dixon (PD‐Dixon). Finally, seven patients (age 62 ± 14 years, 4 males) were scanned using LGE‐Dixon and conventional LGE. The scan time, intraobserver and interobserver variability, and levels of agreement were assessed.

**Statistical Tests:**

Student's *t*‐test, one‐way ANOVA, and Mann–Whitney *U*‐test were used; *P* < 0.05 was considered significant, intraclass correlation coefficient (ICC).

**Results:**

The scan time (minutes:seconds) for LGE‐Dixon (*n* = 28) was 5:01 ± 1:40. ICC values for intraobserver and interobserver measurements of LA wall fibrosis percentage were 0.98 (95% CI, 0.97–0.99) and 0.97 (95% CI, 0.94–0.99) while of EAT were 0.92 (95% CI, 0.82–0.97) and 0.90 (95% CI, 0.80–0.95). The agreement for LA fibrosis percentage between the LGE‐Dixon and the conventional LGE was 0.92 (95% CI, 0.66–0.99) and for EAT volume between the LGE‐Dixon and the PD‐Dixon was 0.93 (95% CI, 0.72–0.98).

**Conclusion:**

LA fibrosis and EAT can be assessed simultaneously using LGE‐Dixon. This method allows a high level of intraobserver and interobserver repeatability as well as agreement with reference methods and can be performed in a clinically feasible scan time.

**Evidence Level:**

2

**Technical Efficacy Stage:**

3

Cardiac MRI is a powerful tool to identify structural changes in atrial tissue and has contributed to the understanding of pathophysiology and progression of atrial fibrillation (AF).^1^ Epicardial adipose tissue (EAT) around the left atrium (LA) can be visualized with cardiac MRI and appears to be independently associated with the incidence, severity, and recurrence of AF.^2^ There appears to be a directly proportional relationship of increasing EAT along the continuum of no AF, paroxysmal, persistent, and permanent AF.^3^ EAT serves as a local energy supply and is associated with LA myocardial inflammation.^4^ Obesity is independently associated with new onset, postoperative, and postablation AF, but the strength of associations of AF with EAT is greater than for measures of abdominal or overall adiposity.^5^, ^6^, ^7^

The hypothesis that EAT may facilitate paracrine effects on the atrial myocardium, by producing adipokines, chemokines, inflammatory cytokines, and growth factors, which can freely diffuse into the adjacent myocardium and may result in fibrotic changes, has been supported by multiple studies.^8^, ^9^, ^10^, ^11^, ^12^, ^13^ The histological study by Haemers et al strongly supports the concept of EAT substrate progression in AF by inflammation‐induced fibrosis, a process in which cytotoxic lymphocytes might be involved.^4^ Fibrosis is the hallmark of atrial structural remodeling, resulting from an excessive deposition of extracellular matrix to replace degenerating atrial myocytes.^14^ Fibrotic tissue can be quantified using late gadolinium enhancement (LGE) MRI and is a major risk factor for AF progression.^15^

Previous works have shown that the degree and spatial distribution of LA fibrosis and EAT can predict the success of AF treatment.^16^, ^17^, ^18^ This pathophysiological relationship and the universal interest for efficient AF‐treatment pathways motivate the exploration of simultaneous assessment of these two AF risk factors.

Combined LGE and Dixon water–fat separation have been proposed to simultaneously visualize fibrosis and fat infiltration in the left ventricle (LV) or to improve fat suppression in LGE.^19^, ^20^, ^21^, ^22^, ^23^ Shaw et al applied LGE‐Dixon in AF patients and found good agreement with the conventional LGE for LA fibrosis visualization, although LA EAT was not considered.^21^

In this study, we sought to perform a comprehensive evaluation on whether LA fibrosis and EAT can be simultaneously assessed using LGE‐Dixon by analysis of repeatability of both fibrosis and EAT quantification as well as systematic comparison with reference methods for assessment of fibrosis and fat.

## Methods

### Patients

**FIGURE 1:**
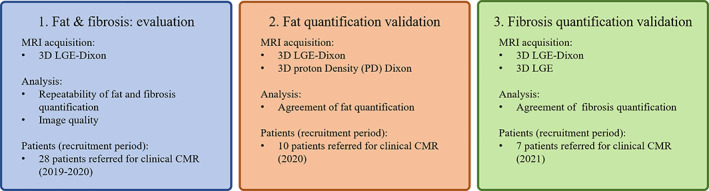
Overview of the imaging protocols, analysis, and patient recruitment of the three substudies. LGE = late gadolinium enhancement.

This prospective study was approved by the institutional review board and all participants provided written informed consent. The study was divided into three substudies (Fig. 1): evaluation of simultaneous assessment of EAT and LA fibrosis, validation of EAT quantification, and validation of fibrosis quantification. First, patients were scanned between February 2019 and February 2020 to evaluate fibrosis and EAT quantification as well as image quality. The second substudy aimed to validate EAT quantification for which patients were recruited between September 2020 and October 2020. The third and last substudy aimed to validate fibrosis quantification for which patients with history of AF were scanned between May 2021 and September 2021. Inclusion criteria: clinical indications for cardiac MRI for all patients and, in addition, history of AF for patients in the third substudy. Exclusion criteria: claustrophobia for all patients in the three substudies. The clinical characteristics of included patients are summarized in Table 1 and their cardiovascular background is reported in Table E1 in the Supplemental Material.

**TABLE 1:** Clinical Characteristics of all 45 Patients Included in the Study

	Evaluation of LGE‐Dixon	Validation of EAT Quantification	Validation of LA Fibrosis Quantification
*N*	28	10	7
Age, years	58 ± 19	46 ± 19	62 ± 14
Male sex, *n* (%)	15 (54)	9 (90)	4 (57)
BMI, kg/m^2^	25 ± 4	27 ± 5	25 ± 4
BSA, m^2^	1.92 ± 0.2	2 ± 0.2	1.91 ± 0.2
Heart rate, beats/minute	71 ± 16	71 ± 11	65 ± 16
SR during scan, *n* (%)	21 (75)	10 (100)	4 (57)
LVEDVI, mL/m^2^	92 ± 25	92 ± 23	92 ± 23
LVEF, %	54 ± 13	54 ± 7	51 ± 12
LA volume, mL	121 ± 52	103 ± 28	151 ± 45
LA volume index, mL/m^2^	62 ± 24	50 ± 13	80 ± 22

LGE = late gadolinium enhancement; EAT = epicardial adipose tissue; BMI = body mass index; BSA = body surface area; LVEDVI = left ventricular end‐diastolic volume index; LA = left atrial; SR = sinus rhythm; LVEF, left ventricular ejection fraction.

### Cardiac MRI Protocol

All experiments were performed on a 1.5‐T scanner (Philips Healthcare, Best, The Netherlands) using a 28‐channel cardiac coil. LGE‐Dixon was performed approximately 20 minutes after gadolinium‐based contrast agent injection (0.2 mmol/kg gadobutrol) with the following imaging parameters: orientation = transverse; field‐of‐view (FOV) = 320 × 320 × 120–140 mm^3^; spatial resolution = 1.25 × 1.25 × 2.5 mm^3^; flip angle = 20°; repetition time (TR)/echo time (TE)_1_/TE_2_ = 7.1/2.2/4.8 msec; pixel bandwidth = 433 Hz; acquisition window = 100 msec, linear profile order. For image acceleration, an online commercial compressed SENSE technique was used with acceleration factor 5.^24^ Water and fat images were reconstructed online using a commercially available modified Dixon (mDIXON) technique to generate in‐phase and out‐of‐phase images.^25^ A diaphragmatic respiratory navigator with 6 mm gating window was used with a restore pulse optimized to minimize inflow artifacts.^26^ Imaging every other heartbeat increases contrast and improves robustness to heart rate variability due to the near complete recovery of longitudinal magnetization; therefore, in patients with sinus rhythm, the inversion pulse was performed every RR‐interval, and for patients in AF during the scan the inversion pulse was performed every two RR‐intervals.^27^ A Look‐Locker was acquired prior to the LGE‐Dixon scan to determine the optimal inversion time which ranged from 220 msec to 340 msec. No arrhythmia rejection algorithms were used. With these imaging parameters, the nominal scan time was 2 minutes and 15 seconds for patients in sinus rhythm and double that for patients with arrhythmia during the scan. The inversion delay was determined using a Look‐Locker scan to null healthy myocardium, and the acquisition was triggered to atrial diastole defined by a time‐resolved four‐chamber image. In the first substudy, patients were scanned with the 3D LGE‐Dixon sequence. The quantified parameters were LA volume (mL), LA fibrosis (% of the LA wall surface), EAT (mL), left ventricular end‐diastolic volume (LVEDV), and ejection fraction (EF), the last two ones by using short‐axis cine balanced steady‐state free precession (bSSFP).

In the second substudy, the 3D proton density Dixon (PD‐Dixon) was acquired and used as a reference for the fat quantification. LGE‐Dixon was performed as described previously, while PD‐Dixon was performed immediately after contrast administration when there was a gap in the CMR protocol to avoid increasing the overall examination duration. PD‐Dixon had the same imaging parameters as LGE‐Dixon, except for the inversion pulse which was removed. For this second cohort, we quantified LA volume (mL) and EAT (mL) using the two Dixon techniques and LVEDV and EF using the short‐axis cine bSSFP.

The proposed 3D LGE‐Dixon and the conventional 3D LGE were performed in the third sub‐study to compare fibrosis area. The imaging parameters for the conventional LGE were FOV = 320 × 320 × 120–140 mm^3^; resolution = 1.25 × 1.25 × 2.5 mm^3^; TR/TE = 4.8/2.4 msec; pixel bandwidth = 360 Hz; acquisition window = 100 msec, centric profile order. Conventional SENSE was used for image acceleration with a factor of 2 in the phase encoding direction, and identical respiratory motion compensation as for 3D LGE‐Dixon. The order of the 3D LGE‐Dixon and the standard 3D LGE was randomized. In this last cohort, we quantified LA volume (mL) and LA fibrosis (% of the LA wall surface) using the two LGE techniques and LVEDV and EF using the short‐axis cine bSSFP.

### Image Analysis

Water–fat separated images were reconstructed on the scanner using vendor‐provided Dixon and compressed SENSE algorithms. The water images were used for LGE quantification, while the fat images were used to quantify the EAT.^28^, ^29^ Segmentations of the LA myocardium and EAT in the first substudy (*n* = 28) were performed twice by reader 1‐IS (2 years of cardiovascular imaging experience), with at least 2 week's time between the segmentations, for intraobserver variability analysis and once by reader 2‐MH (12 years of cardiac MRI experience) for interobserver variability analysis. Two additional readers, 3‐SS (less than 1 year of cardiovascular imaging experience) and 4‐JS (3 years of cardiovascular imaging experience), performed the segmentations of the 19 of these 28 patients who had prior or present cardiovascular disease. LA myocardium and fat segmentation were performed using the Medical Imaging Interaction Toolkit (MITK, German Cancer Research Center, Heidelberg, Germany), while automatic fibrosis and fat quantification were implemented on a commercial workstation using MATLAB R2021b (The MathWorks, Natick, MA, USA). The 3D LA myocardium segmentation consisted of manual tracing of the left atrial wall (intramural) and exclusion of the left atrial appendage, left and right pulmonary veins antrums, and mitral valve. The automatic fibrosis quantification was performed by obtaining a patient‐specific threshold, based on the signal intensity (SI) of the mitral valve.^30^, ^31^ Figure S1 in the Supplemental Material shows an example mitral valve segmentation, which is used together with the LA blood pool SI to obtain a patient‐specific threshold.

**FIGURE 2:**
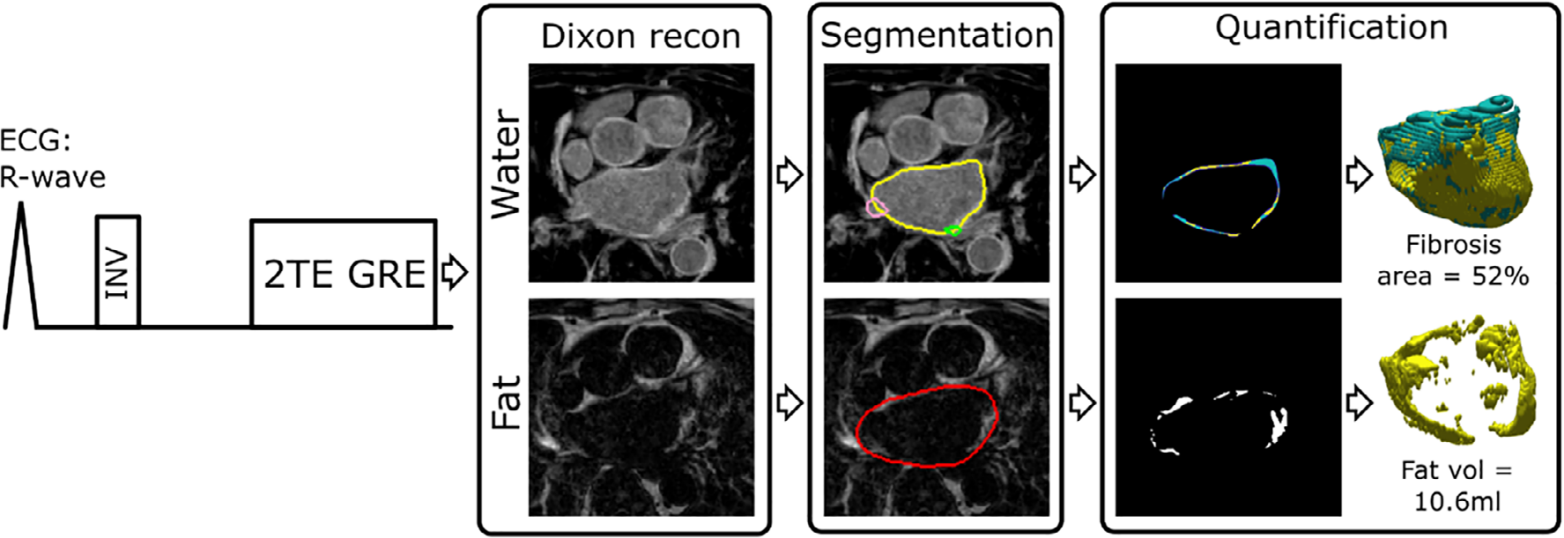
Acquisition, reconstruction, segmentation, and quantification pipeline of the proposed late gadolinium enhancement (LGE)‐Dixon. An electrocardiogram (ECG)‐triggered inversion‐recovery (INV) pulse sequence was used with dual echo gradient echo (2TE GRE) readout to enable Dixon reconstruction and water–fat separation. The water images allowed left atrial (LA) wall segmentation where the right and left pulmonary veins, left atrial appendage, and mitral valve were excluded from the LA wall segmentation. An automated method was used to classify the LA wall tissue as healthy or fibrotic. The LA scar area was calculated as the number of scar pixels divided by the total number of LA wall pixels. A separate fat segmentation was used to delineate and quantify the (epicardial adipose tissue) EAT. LGE‐Dixon was acquired in an axial orientation, approximately 20 minutes after gadolinium‐based contrast agent injection. The LGE‐Dixon images are from a 79‐year‐old woman with ongoing atrial fibrillation during the scan.

Dixon fat images were used for EAT segmentation and quantification. The EAT outer border was manually traced by all readers, on consecutive transaxial slices, while the inner border was obtained by using the LA intramural segmentation. Interatrial septal fat and adjacent right atrial fat was included in the segmentation. The LA segmentation used the mitral annulus as inferior border and bifurcation of the pulmonary artery as upper border. The procedure for segmentation of all included LA structures in one patient is illustrated in Fig. S2 in the Supplemental Material. Example LGE‐Dixon images from one patient, including LA myocardium and EAT segmentation and quantification are shown in Fig. 2 (image acquisition during AF). Fibrosis is reported as percentage of the total atrial wall surface.

Visual scoring was performed separately for the LGE and EAT images in the 28 patients who were initially scanned by two readers (reader 5‐CC with more than 20 years of experience in cardiac imaging and reader 1) to assess image quality. A 5‐point Likert‐type scale, similar to Gunasekaran et al, was used by both readers to grade key features such as the LA wall, paracardial fat, and proximal coronary arteries in the images according to the following criteria: 1 = nondiagnostic, 2 = moderate, 3 = good, 4 = very good, and 5 = excellent image quality. Prior to visual evaluation, the readers were given training data with examples of LGE and EAT images with nondiagnostic to excellent quality to calibrate readers' scores.^32^ Example images for each visual score category that were provided to the readers before the scoring are shown in Fig. S3 in the Supplemental Material. Because image quality can be affected by arrhythmia, we compared visual scores in nine patients with irregular heart rates (six patients with AF and three with other arrhythmia; age = 67 ± 13 years; six males) to nine age and sex matched patients in sinus rhythm (age = 64 ± 13 years; six males).

### Statistical Analysis

Continuous variables are expressed as mean ± SD. Non‐normal distributed data are expressed as median (interquartile range). Categorical variables are expressed as counts and percentages. Student's *t*‐test and one‐way analysis of variance (ANOVA) were used for normal distributed variables, while Mann–Whitney *U*‐test was used for non‐normal distributed variables. All tests were two sided and a *P* < 0.05 was considered statistically significant. Intraobserver and interobserver reliability measurements and agreement between reference and proposed methods were assessed with the intraclass correlation coefficient (ICC). ICC estimates and their 95% confident intervals were calculated using absolute‐agreement two‐way mixed‐effects model, single rater/measurement.^33^ Bland–Altman analysis was used for the agreement between EAT quantification with LGE‐Dixon and PD‐Dixon as well as fibrosis quantification with LGE‐Dixon and conventional Dixon. Statistical analysis was performed using IBM SPSS Statistics for Windows, version 28.0 (IBM Corp., Armonk, NY, USA).

## Results

### Populations

We scanned 28, 10, and 7 patients with clinical indications for cardiac MRI. The baseline characteristics are summarized in Table 1. Notably, in the EAT validation group, the majority were men (9/10) and they had significantly smaller LA volume than the LA fibrosis validation group. All MRI scans were successfully completed.

### LGE‐Dixon for Simultaneous Visualization and Analysis of LA Fibrosis and EAT

**FIGURE 3:**
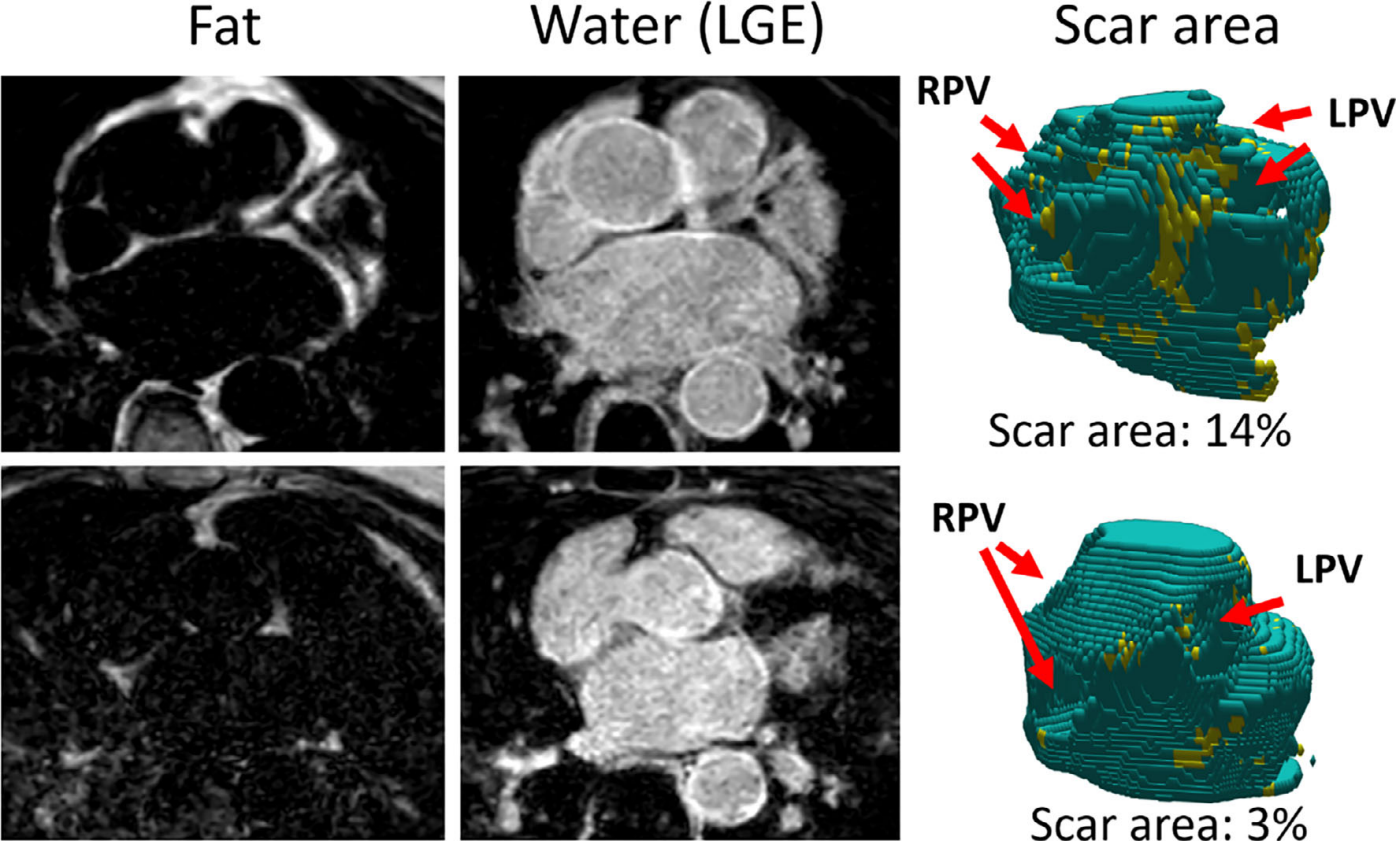
Late gadolinium enhancement (LGE)‐Dixon images (acquired in an axial plane) and fibrosis quantification in one patient with atrial fibrillation (AF, top row) and in sinus rhythm (bottom row). Increased area of fibrosis, indicated by the yellow regions in the volume rendered left atrial (LA) to the right and quantified as the percentage of the total LA surface, was measured in the AF patient compared to the patient in sinus rhythm. The fat and LGE images for both patients were visually scored as having a good image quality (visual score = 3). RPV = right pulmonary veins; LPV = left pulmonary veins.

**FIGURE 4:**
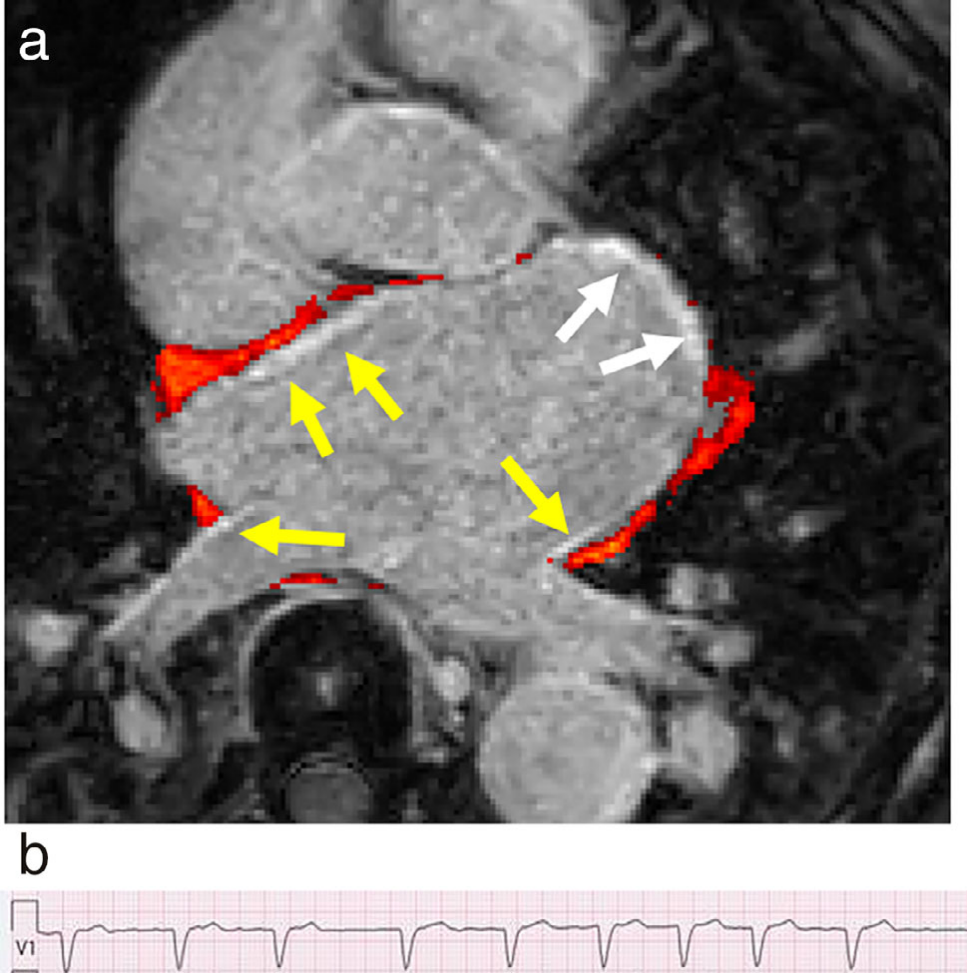
(**a**) Transaxial midatrial image showing fused late gadolinium enhancement (LGE) and epicardial adipose tissue (EAT) scans from one patient with ongoing atrial fibrillation (69‐year‐old male), demonstrating notable spatial correlation between fibrosis and EAT. The red surfaces represent EAT. The yellow arrows highlight LGE of the left atrial wall and pulmonary ostia with adjacent EAT. The white arrows indicate the mitral valve. LGE‐Dixon was performed approximately 20 minutes after gadolinium‐based contrast agent injection. (**b**) ECG‐strip confirming atrial fibrillation.

The first scanned cohort (28 patients, 15 males) had a mean scan time (in minutes:seconds) ± SD of 5:01 ± 1:40. Nineteen patients had sinus rhythm (SR) during the cardiac MRI registration and nine had AF or other arrhythmia. Representative images for one patient with AF and one patient with SR are shown in Fig. 3. Fused LGE and EAT images from one patient with AF are shown in Fig. 4, demonstrating high spatial correlation between fibrosis and EAT in this patient.

#### ***LA*** ***Fibrosis Area and LA EAT Volume***

The LA fibrosis measurements as percentage of the total LA surface area were 9.4 (11.6) %. The mean EAT value was 13 ± 10 mL, and the mean of body surface area (BSA) indexed EAT was 6.5 ± 4.5 mL/m^2^. The ICC values for interobserver and intraobserver measurements of LA volume, LA fibrosis, and EAT were good to excellent and are summarized in Table 2, both for two readers (28 patients) and four readers (19 patients)

**TABLE 2:** . Interobserver and Intraobserver Variability Measurements

	Interclass Correlation Coefficient	
	Interobserver	Intraobserver
Two Readers (28 patients)		
LA volume, mL	0.98 (95% CI, 0.95–0.99)	0.99 (95% CI, 0.98–0.99)
EAT, mL	0.90 (95% CI, 0.80–0.95)	0.92 (95% CI, 0.82–0.97)
LA fibrosis, %	0.97 (95% CI, 0.94–0.99)	0.98 (95% CI, 0.97–0.99)
Four readers (19 patients)		
LA volume, mL	0.97 (95% CI, 0.93–0.99)	
EAT, mL	0.77 (95% CI, 0.60–0.90)	
LA fibrosis, %	0.96 (95% CI, 0.91–0.98)	

LA = left atrial; EAT = epicardial adipose tissue; CI = confidence interval.

#### Image Quality Assessment

The median visual score (25th and 75th percentile) for all 28 patients was 3 (3, 4) both for the LGE and EAT image quality assessment. Furthermore, all images were of diagnostic quality (visual score > 1). The distribution of visual scores for the 28 patients, including both LGE and EAT, are shown in Fig. S4 in the Supplemental Material. The median LGE image quality for the arrhythmic patients was 3 (3, 4) and for the patients in SR 4 (3, 5) (*P* = 0.23). The median EAT image quality for the arrhythmic patients was 3 (3, 4) and for the patients in SR 4 (3, 5) (*P* = 0.12). The scan time for the arrhythmic patients (6:29 ± 1:33), where inversion pulse was performed every two heartbeats, was significantly longer than for SR patients (4:54 ± 1:21) where the inversion was performed every heartbeat.

### Eat Quantification Using LGE‐Dixon and PD‐Dixon

**FIGURE 5:**
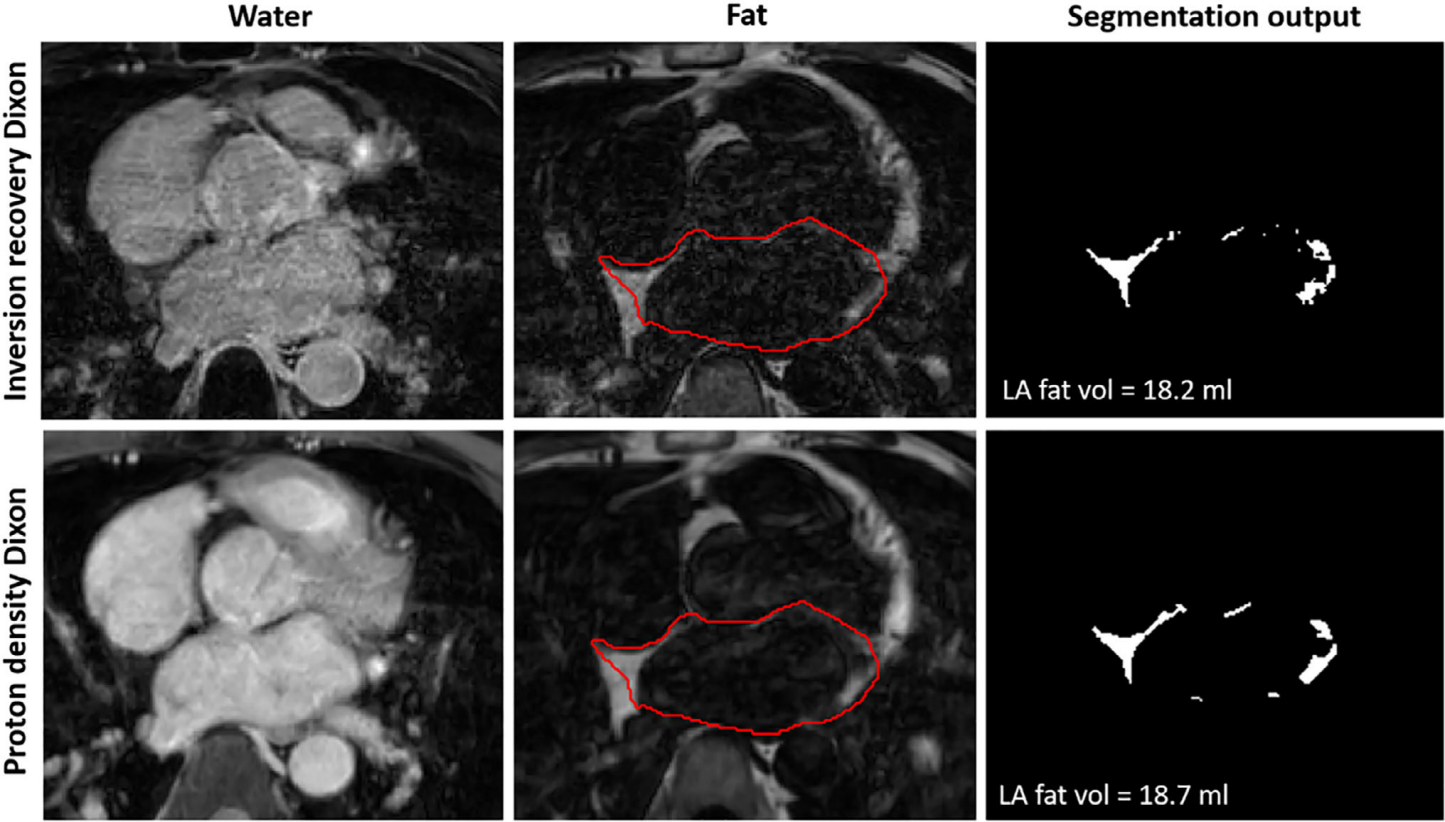
Example axial images of reconstruction, segmentation, and quantification of epicardial adipose tissue in late gadolinium enhancement (LGE)‐Dixon (top row) and proton density (PD)‐Dixon (bottom row) scans from the same patient (51‐year‐old male, scan during sinus rhythm). PD‐Dixon was performed immediately after gadolinium‐based contrast agent injection, while LGE‐Dixon was performed approximately 20 minutes after contrast agent injection. LA = left atrial.

**FIGURE 6:**
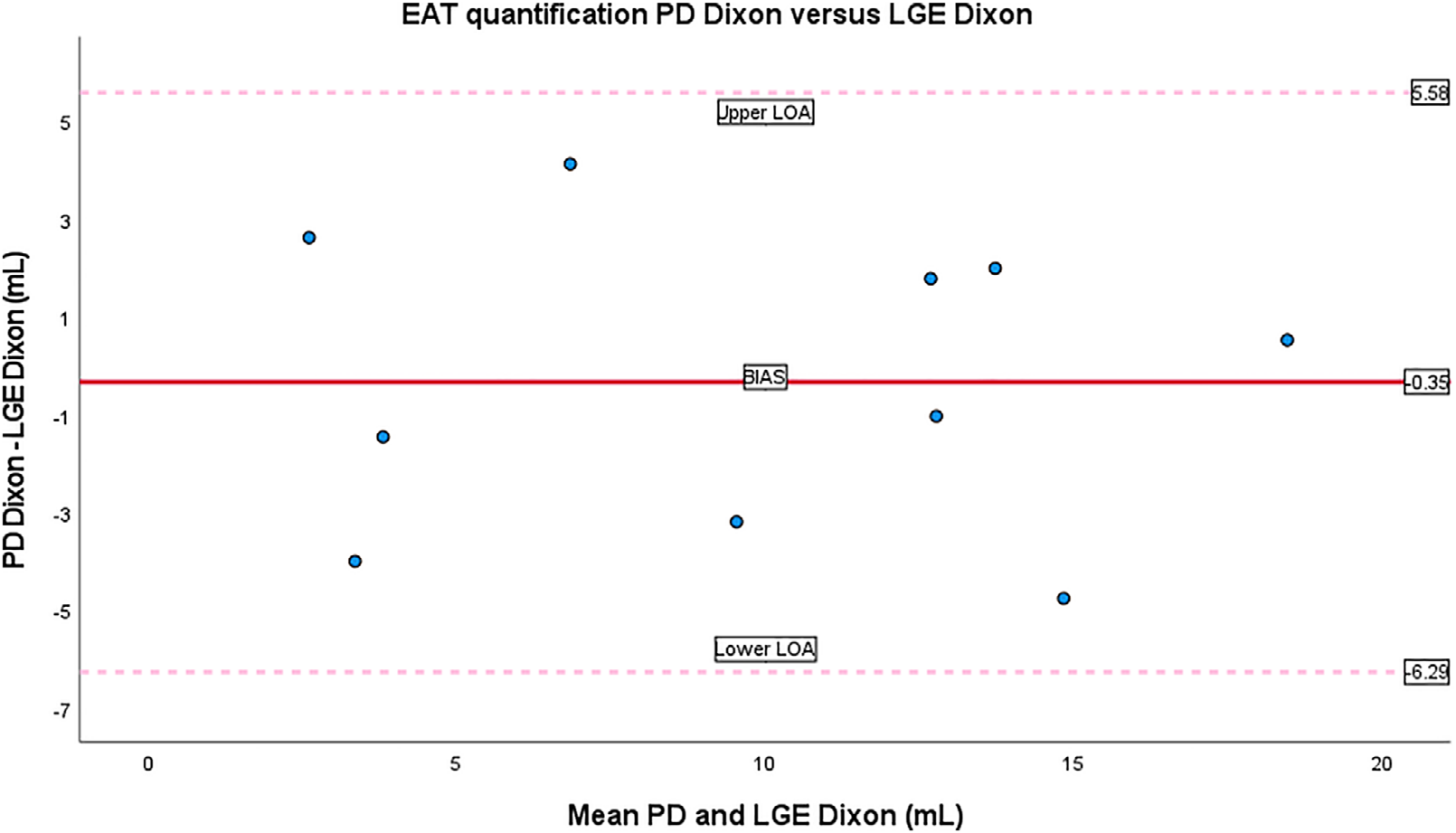
Bland–Altman plots of agreement between proton density (PD) Dixon and late gadolinium enhancement (LGE)‐Dixon. The lines represent the mean difference (bias = −0.35) and the 95% limits of agreement (upper limits of agreement [LOA] = 5.58, lower LOA = −6.29, standard deviation [SD] = 3.03). *Y*‐axis: Difference of corresponding values in milliliter; *X*‐axis: Mean of corresponding values in milliliter. EAT = epicardial adipose tissue.

LGE‐Dixon and PD‐Dixon scans were performed in the second group of 10 patients. The scan times of the PD‐Dixon and 3D LGE‐Dixon were 4:28 ± 0:43 and 4:29 ± 0:55, respectively. Example images for the LGE‐Dixon and PD‐Dixon scans in the same patient are shown in Fig. 5. The patient heart rate during the LGE‐Dixon scan was 71 ± 11 beats/minute and for the PD‐Dixon scan 74 ± 11 beats/minute, a difference that was statistically significant. The BSA‐indexed EAT was 4.9 ± 2.6 mL/m^2^ according to LGE‐Dixon and 4.7 ± 2.6 mL/m^2^ according to PD‐Dixon (*P* = 0.70). The level of agreement between the LGE‐Dixon and PD‐Dixon methods was excellent (ICC = 0.93, [95% CI, 0.72–0.98]). The Bland–Altman plot (Fig. 6) confirms this finding.

### LA Fibrosis Quantification Using 3D LGE‐Dixon and Conventional LGE

**FIGURE 7:**
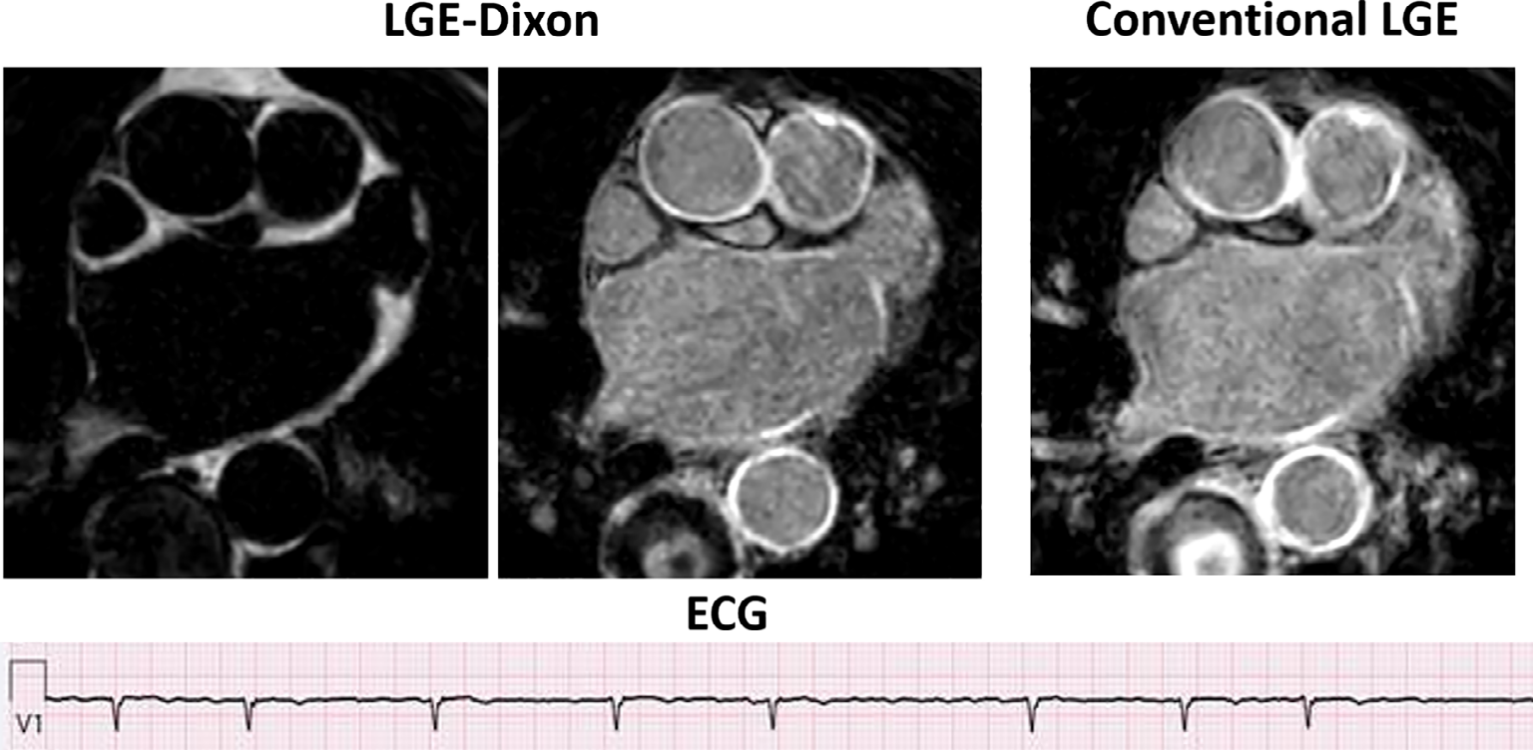
Example axial images of late gadolinium enhancement (LGE)‐Dixon and conventional LGE scans from a 77‐year‐old patient during atrial fibrillation. ECG‐strip confirming atrial fibrillation.

**FIGURE 8:**
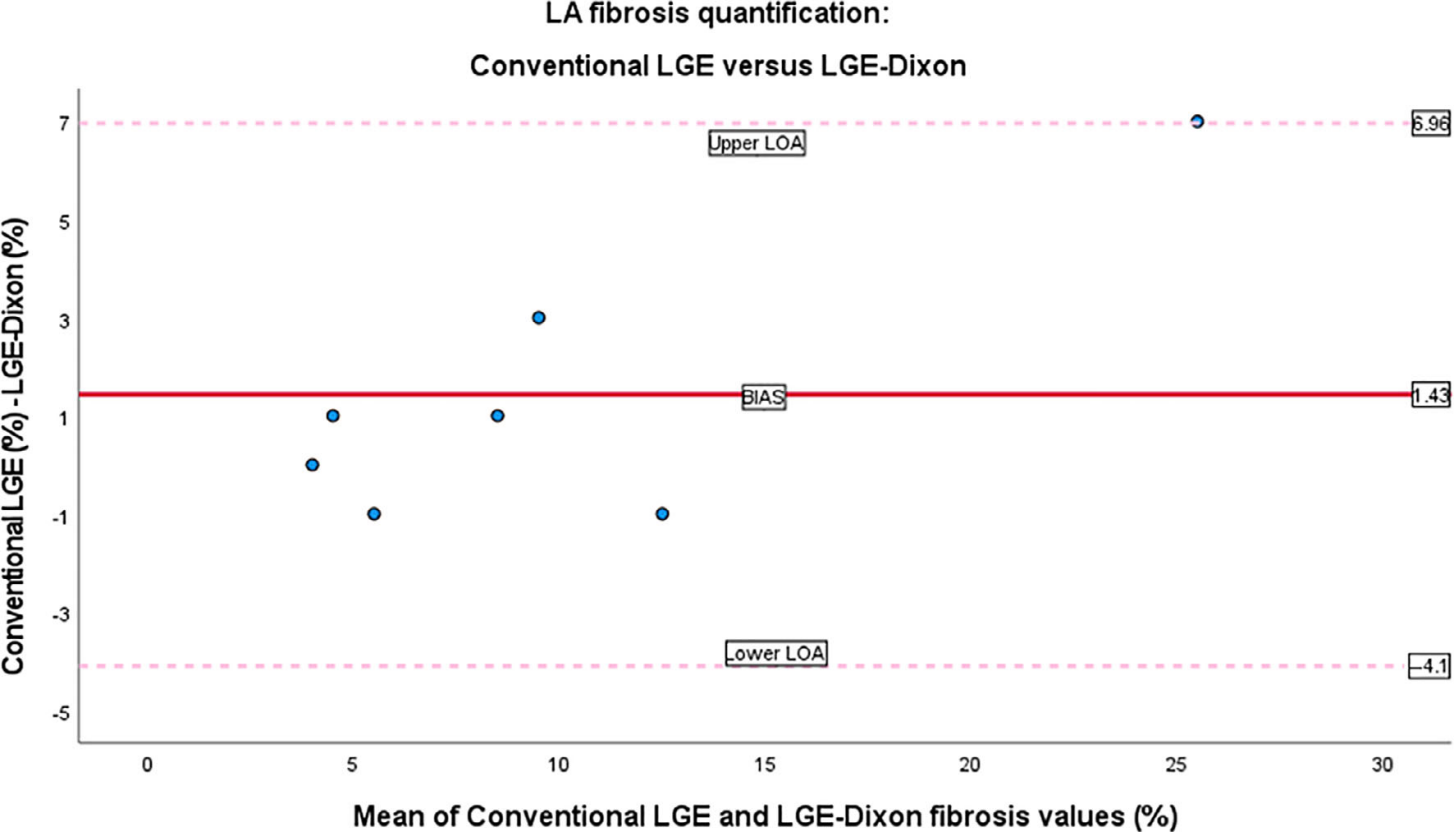
Bland–Altman plots of agreement between conventional late gadolinium enhancement (LGE) and LGE‐Dixon. The lines represent the mean difference (bias = 1.43) and the 95% limits of agreement (lower limits of agreement [LOA] = −4.1, upper LOA = 6.96, standard deviation [SD] = 2.82). *Y*‐axis: Difference of relative fibrosis are values in percentage; *X‐*axis: Mean of relative fibrosis area values in percentage. LA = left atrial.

The substudy comparing 3D LGE‐Dixon and conventional LGE in terms of LA fibrosis consisted of seven individuals with history of AF. Three of these seven patients were in AF during the cardiac magnetic resonance (CMR) scan. The scan time for LGE‐Dixon was 8:05 ± 2:56, significantly shorter than for conventional LGE which was 12:02 ± 4:45. Example images for the LGE‐Dixon and the conventional LGE from a patient with ongoing AF are shown in Fig. 7. Patient heart rate during the LGE‐Dixon scan was 65 ± 16 beats/minute and for the conventional LGE scan 66 ± 17 beats/minute (*P* = 0.54). The measured fibrosis area reported as median (interquartile range) was 8 (9)% using LGE‐Dixon and 9 (7)% using conventional LGE (*P* = 0.24). The level of agreement between the LGE‐Dixon and conventional LGE methods was excellent (ICC = 0.92 [95% CI, 0.66–0.99]). The Bland–Altman plot (Fig. 8) confirms this finding.

## Discussion

A comprehensive evaluation of 3D LGE‐Dixon MRI was performed. Simultaneous visualization and quantification of LA LGE and EAT is feasible using LGE‐Dixon. Furthermore, the technique provides excellent repeatability of both LA fibrosis and EAT and agreement with conventional methods for assessment of fibrosis and fat.

The combined use of Dixon and LGE has been hampered in the past by the increased scan time required to obtain multiple echoes. Conventionally, parallel imaging has been used with an acceleration factor of 2, while more recent compressed sensing techniques have used factors of 3 and 3.3, respectively.^32^, ^34^ In this study, compressed sensing was employed with a factor 5 to maintain a clinically acceptable scan time of approximately 5 minutes (including respiratory gating). Even in patients with arrhythmia, where the inversion pulse and imaging were performed every two heartbeats, the scan time was still reasonable (between 6 and a ½ minutes in substudy 1 and 8 minutes in substudy 2) due to the high acceleration factor. Importantly, there were no statistically significant differences in subjective image quality between arrhythmic patients and patients in SR. Imaging every other heartbeat likely contributed to good image quality in this often‐challenging patient cohort. These results warrant further studies of 3D LGE‐Dixon in patients with AF where long scan times and poor image quality has hampered the use of 3D LGE in the past. As noted in the previous studies using LGE‐Dixon, an advantage of this approach compared to the conventional LGE is the ability to flexibly eliminate fat signal from the LGE images. Fibrosis and fat have a similar SI using T_1_‐weighted gradient‐echo readout. Without suppression, fat may be misinterpreted as fibrosis, a particular concern for visualizing fibrosis in the LA which is often adjacent to EAT. Fat suppression pulses are typically used for LA LGE to avoid this. However, EAT contains clinically relevant information which can be captured using a multiecho readout to complement the simultaneously acquired fibrosis information. In the context of fat suppression/separation, 3D LGE‐Dixon allows the use of linear *k*‐space acquisition order which improves contrast and image sharpness and also avoids blurring compared to centric order required for fat suppression pulses.

Evaluation of fibrosis and fat could be performed using CMR but without the combined LGE‐Dixon approach proposed in this work. This would involve performing PD‐Dixon as a separate scan to obtain LA EAT information and conventional LA LGE for fibrosis. However, this would have several drawbacks: firstly, the combined scan time would be significantly longer, as indicated in this study it would require two scans, each of 4–10 minutes in duration, whereas LGE‐Dixon only requires one. Second, the LA EAT and fibrosis would not necessarily be spatially aligned and therefore require additional image registration to allow analysis of fat and fibrosis co‐localization, whereas for LGE‐Dixon the fat and LGE images are intrinsically spatially aligned.

In a previous study, Nakamori et al assessed LA EAT volume in patients with and without a history of AF using a 3D multiecho Dixon fat–water separated MRI sequence.^29^ Repeatability analysis was performed in 10 AF patients and showed an agreement for both interobserver and intraobserver analysis that was comparable to the findings in this study. Repeatability analysis of the LA enhancement on LGE‐Dixon images in 12 AF patients was performed by Shaw et al, who found a moderate intraobserver agreement.^21^

The use of LA 3D LGE‐Dixon has been explored in several studies in recent years. This has primarily involved feasibility studies and technical developments such as combining it with advanced respiratory motion compensation and compressed sensing.^21^, ^22^ Another study sought to improve fat visualization by using a water selective inversion pulse, although quantification was not considered.^35^, ^36^ Recent evidence highlighting the role of LA EAT as a possible cause for fibrosis and AF justifies further development of LGE‐Dixon to elucidate the relationship between fibrosis and EAT in patients with AF.^37^ This study shows that LA fibrosis and EAT can be reliably and reproducibly quantified using 3D LGE‐Dixon. However, advanced motion compensation or water selective inversion was not included in this work but may further improve the technique.

The available literature describes several methods for fibrosis quantification.^38^ In this work, the SI from the mitral valve was used as a threshold to quantify scar. This method performs favorably compared to other techniques and was considered an attractive option for quantification in the patients where 3D LGE was performed twice (to compare conventional LGE versus LGE‐Dixon) as it is less dependent on the signal in the blood pool and is easy to implement.^30^ However, alternative quantification techniques will be considered in future work which may provide even more robust fibrosis quantification.

Large areas of LGE were found in five patients with cardiac amyloidosis. The extensive LGE involvement of the LA myocardium, in combination with a marked reduction of LA contractile function, is a prevalent feature of patients with cardiac amyloidosis characterized by cardiac MRI.^39^ Therefore, the high values described in this study are not unusual.

The current LGE‐Dixon approach allows spatial co‐localization of LA wall fibrosis and EAT and this ability can provide new perspectives on the coupling and interplay between these two pathophysiological factors in development and progress of AF. The simultaneous visualization of LA fibrosis and EAT, alongside the other parameters of LA and LV structure and function, allows a comprehensive patient risk assessment both before and after the development of atrial arrhythmia.^17^, ^31^ Furthermore, the spatial distribution of EAT and fibrosis can guide the AF ablation.^17^, ^18^ Future work will be focused on quantitative comparison between LA fibrosis and EAT in terms of, eg, spatial co‐localization and amount of tissue in patients with paroxysmal AF and persistent AF.

### Limitations

A relatively small number of patients was included in this single‐center, single‐magnet study, although it is comparable to similar method development studies. Further studies are ongoing in a more homogeneous and larger cohort, focusing on patients with different types of AF. A technical limitation of the LGE‐Dixon technique is that the fat signal will have a lower signal‐to‐noise ratio than proton density weighted Dixon due to the effects of the inversion pulse. Theoretically, the fat signal could be nulled by the inversion pulse which would preclude fat quantification. However, the inversion time is set to null healthy myocardium which typically has a much longer postcontrast T1 (approximately 500 msec) than that of fat (T1 = 280 msec, unaffected by the contrast agent). A more probable but undesirable scenario is the water–fat phase wrap which may occur at low signal‐to‐noise ratio.^28^ However, phase wraps were not observed in any of the patients in which LGE‐Dixon was performed, suggesting the phase unwrapping method is sufficiently robust for the proposed pulse sequence. This potential issue could be remedied by using a spectrally selective re‐inversion pulse to restore the fat signal or using a water‐selective inversion pulse.^36^, ^40^ Furthermore, no patient with arrhythmia was included in the population where LGE‐Dixon and PD‐Dixon were compared.

## Conclusions

LA fibrosis and EAT can be assessed simultaneously using LGE‐Dixon. This method allows a high level of intraobserver and interobserver repeatability as well as agreement with reference methods and can be performed in a clinically feasible scan time.

## Supporting information


**Figure S1** Demonstration of mitral valve (MV) segmentation (purple area) to estimate MV signal intensity (SI) used for fibrosis quantification. The difference between mean MV SI and mean LA blood pool SI was calculated and fibrosis was defined as pixels on the LA wall with SI higher than half the difference + mean LA blood pool SI. The dashed yellow line indicates a profile through the MV and LA blood pool for which the SI is plotted on the right, including the definition of components used for fibrosis SI definition.

**Figure S2** Segmented LA structures in the LGE (top two rows) and fat images (bottom two rows) for two slices in one patient. Segmentations are shown in the second column where the LA wall was segmented in the LGE images (yellow segmentation), in addition to the right pulmonary veins (RPV, green segmentation), left pulmonary veins (LPV, green segmentation) left atrial appendage (AA, green segmentation) and mitral valve (MV, blue segmentation). The overlapping area between the LA and other segmented structures were subtracted to yield the LA wall only (third column). The LA fat segmentation is shown in red. Volumetric representation of the fibrosis and fat quantification are shown on the right.

**Figure S3** Example LGE (top row) and fat (bottom row) images for the different image quality categories used for the visual scoring.

**Figure S4**. Distribution of visual scoring of LGE and EAT image quality for the 28 patients. LGE ‐ Late Gadolinium enhancement; EAT ‐ Epicardial adipose tissue.

**Table E1** – Cardiovascular background of all the 45 patients included in the study.
