# Supplementary figures and images for: Simultaneous Assessment of Left Atrial Fibrosis and Epicardial Adipose Tissue Using 3D Late Gadolinium Enhanced Dixon MRI
Source: J Magn Reson Imaging. 2022 Feb 7;56(5):1393–403. doi: 10.1002/jmri.28100 (PMC9790523; doi:10.1002/jmri.28100)

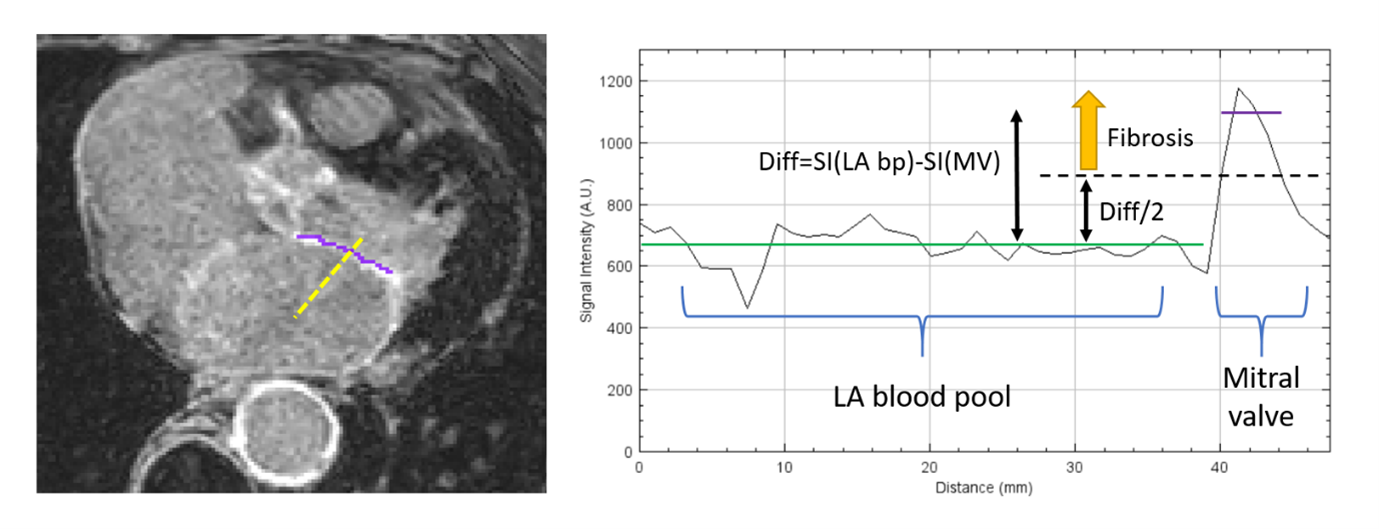

Supplement: Supplementary file 1 — Figure S1 Demonstration of mitral valve (MV) segmentation (purple area) to estimate MV signal intensity (SI) used for fibrosis quantification. The difference between mean MV SI and mean LA blood pool SI was calculated and fibrosis was defined as pixels on the LA wall with SI higher than half the difference + mean LA blood pool SI. The dashed yellow line indicates a profile through the MV and LA blood pool for which the SI is plotted on the right, including the definition of components used for fibrosis SI definition. [file JMRI-56-1393-s002.tif]

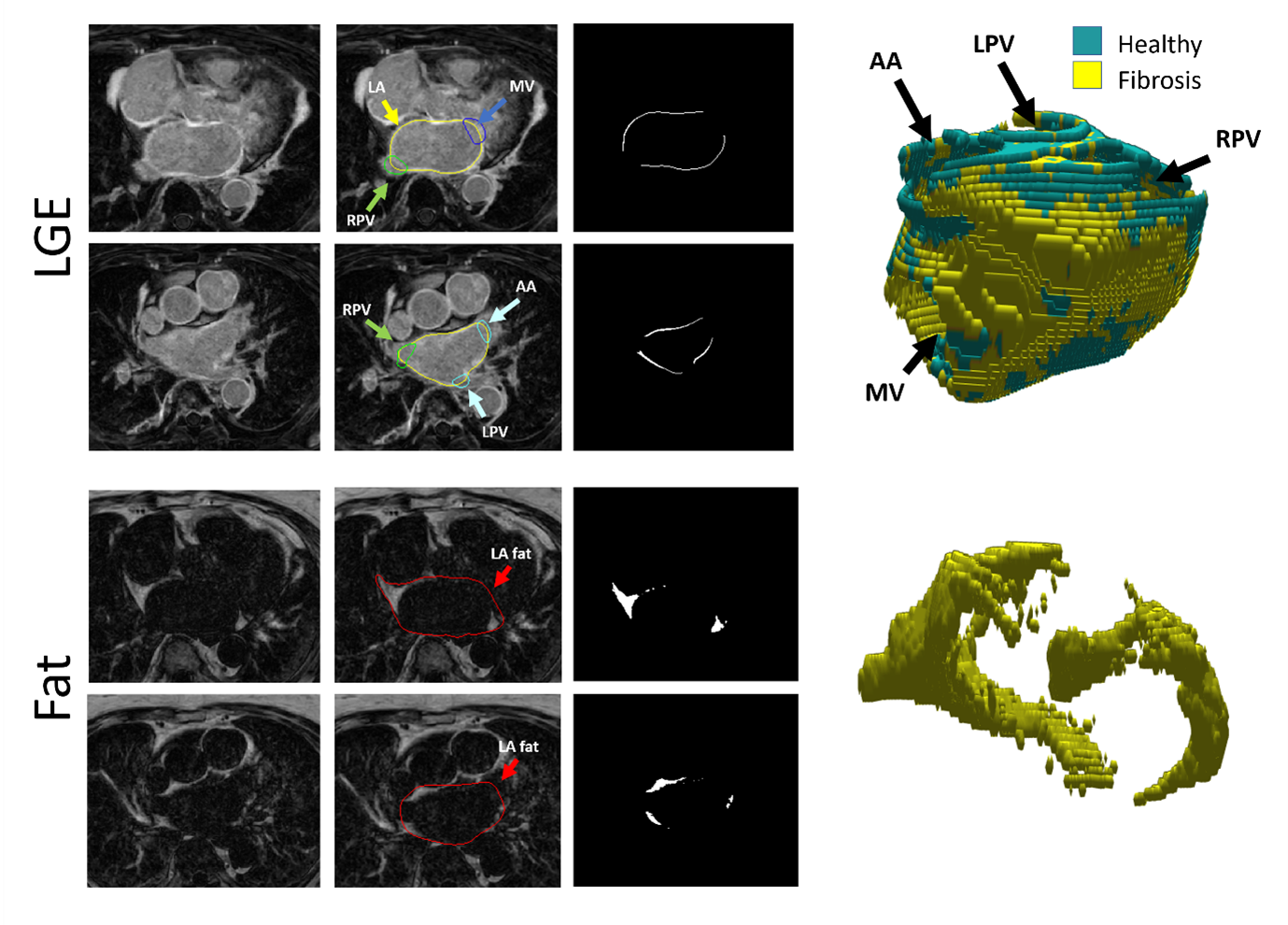

Supplement: Supplementary file 2 — Figure S2 Segmented LA structures in the LGE (top two rows) and fat images (bottom two rows) for two slices in one patient. Segmentations are shown in the second column where the LA wall was segmented in the LGE images (yellow segmentation), in addition to the right pulmonary veins (RPV, green segmentation), left pulmonary veins (LPV, green segmentation) left atrial appendage (AA, green segmentation) and mitral valve (MV, blue segmentation). The overlapping area between the LA and other segmented structures were subtracted to yield the LA wall only (third column). The LA fat segmentation is shown in red. Volumetric representation of the fibrosis and fat quantification are shown on the right. [file JMRI-56-1393-s003.tif]

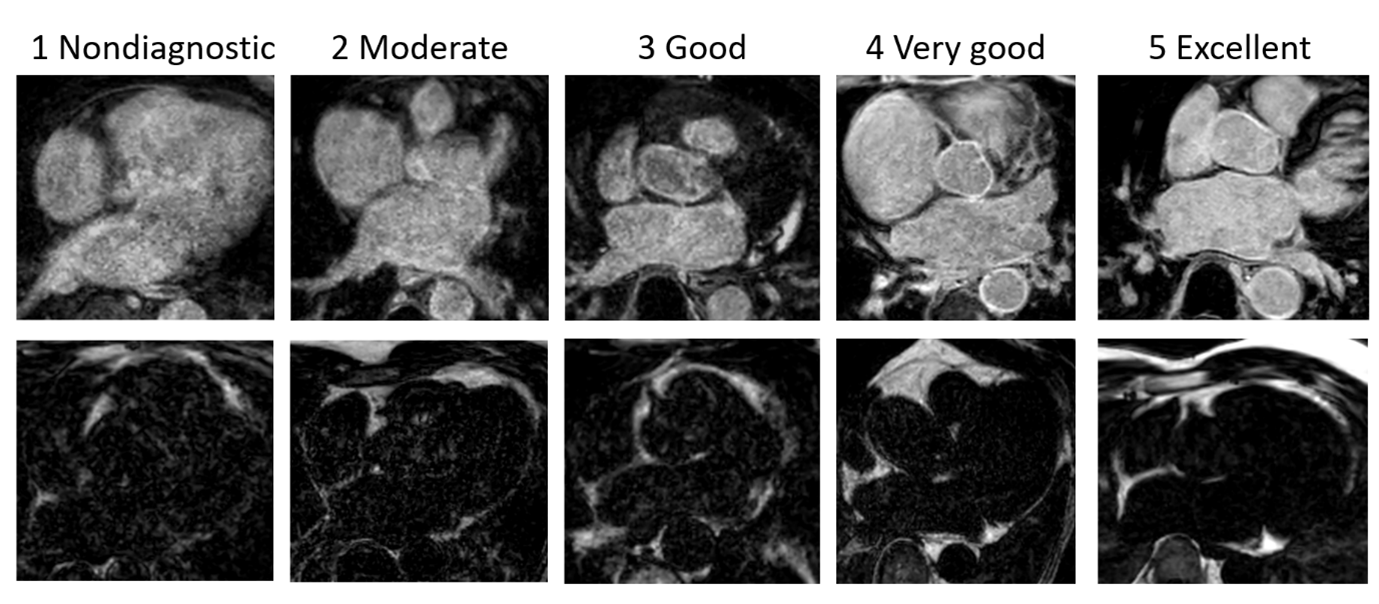

Supplement: Supplementary file 3 — Figure S3 Example LGE (top row) and fat (bottom row) images for the different image quality categories used for the visual scoring. [file JMRI-56-1393-s005.tif]

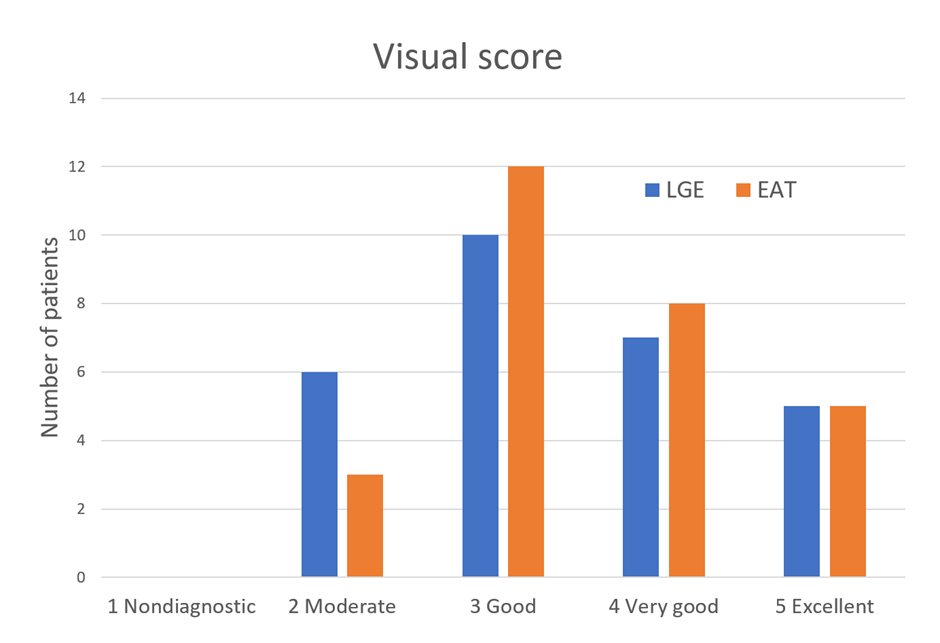

Supplement: Supplementary file 4 — Figure S4. Distribution of visual scoring of LGE and EAT image quality for the 28 patients. LGE ‐ Late Gadolinium enhancement; EAT ‐ Epicardial adipose tissue. [file JMRI-56-1393-s004.tif]
