# Supplementary material for: Simultaneous Assessment of Left Atrial Fibrosis and Epicardial Adipose Tissue Using 3D Late Gadolinium Enhanced Dixon MRI
Source: J Magn Reson Imaging. 2022 Feb 7;56(5):1393–403. doi: 10.1002/jmri.28100 (PMC9790523; doi:10.1002/jmri.28100)
Supplement: Supplementary file 5 — Table E1 – Cardiovascular background of all the 45 patients included in the study. [file JMRI-56-1393-s001.docx]

**Supplementary table E1** - Cardiovascular background of all the 45 patients included in the study

|  | Evaluation of | Validation of EAT | Validation of LA |
| --- | --- | --- | --- |
|  | LGE-Dixon | quantification | fibrosis quantification |
| N | 28 | 10 | 7 |
| Atrial fibrillation | 10 |  | 7 |
| Arterial hypertension | 8 | 3 | 1 |
| Heart failure | 12 |  | 2 |
| Ischemic heart disease | 6 | 3 | 1 |
| Amyloidosis | 5 |  |  |
| Hypertrophic cardiomyopathy | 4 | 1 |  |
| Diabetes mellitus | 1 | 2 |  |
| Myopericarditis |  | 3 |  |
| Sarcoidosis |  | 1 |  |
| Dilated cardiomyopathy | 3 | 1 |  |
| Takotsubo cardiomyopathy | 1 | 1 |  |
| Thalassemia major |  |  | 1 |
| Other arrhythmia |  |  | 2 |
| Stroke |  |  | 1 |
| FH |  |  | 1 |

LGE - Late Gadolinium enhancement; EAT - Epicardial adipose tissue; LA - Left atrial, FH - Familial hypercholesterolemia.
